# Analyzing and predicting the risk of death in stroke patients using machine learning

**DOI:** 10.3389/fneur.2023.1096153

**Published:** 2023-02-03

**Authors:** Enzhao Zhu, Zhihao Chen, Pu Ai, Jiayi Wang, Min Zhu, Ziqin Xu, Jun Liu, Zisheng Ai

**Affiliations:** ^1^School of Medicine, Tongji University, Shanghai, China; ^2^School of Business, East China University of Science and Technology, Shanghai, China; ^3^Department of Computer Science and Technology, School of Electronics and Information Engineering, Tongji University, Shanghai, China; ^4^Department of Industrial Engineering and Operations Research, Columbia University, New York, NY, United States; ^5^Clinical Research Center for Mental Disorders, Chinese-German Institute of Mental Health, Shanghai Pudong New Area Mental Health Center, School of Medicine, Tongji University, Shanghai, China; ^6^Department of Medical Statistics, School of Medicine, Tongji University, Shanghai, China

**Keywords:** stroke, stroke mortality, machine learning, deep learning, treatment heterogeneity

## Abstract

**Background:**

Stroke is an acute disorder and dysfunction of the focal neurological system that has long been recognized as one of the leading causes of death and severe disability in most regions globally. This study aimed to supplement and exploit multiple comorbidities, laboratory tests and demographic factors to more accurately predict death related to stroke, and furthermore, to make inferences about the heterogeneity of treatment in stroke patients to guide better treatment planning.

**Methods:**

We extracted data from the Medical Information Mart from the Intensive Care (MIMIC)-IV database. We compared the distribution of the demographic factors between the control and death groups. Subsequently, we also developed machine learning (ML) models to predict mortality among stroke patients. Furthermore, we used meta-learner to recognize the heterogeneity effects of warfarin and human albumin. We comprehensively evaluated and interpreted these models using Shapley Additive Explanation (SHAP) analysis.

**Results:**

We included 7,483 patients with MIMIC-IV in this study. Of these, 1,414 (18.9%) patients died during hospitalization or 30 days after discharge. We found that the distributions of age, marital status, insurance type, and BMI differed between the two groups. Our machine learning model achieved the highest level of accuracy to date in predicting mortality in stroke patients. We also observed that patients who were consistent with the model determination had significantly better survival outcomes than the inconsistent population and were better than the overall treatment group.

**Conclusion:**

We used several highly interpretive machine learning models to predict stroke prognosis with the highest accuracy to date and to identify heterogeneous treatment effects of warfarin and human albumin in stroke patients. Our interpretation of the model yielded a number of findings that are consistent with clinical knowledge and warrant further study and verification.

## 1. Introduction

Stroke is an acute disorder characterized by dysfunction of the focal neurological system, underlying cerebral vascular spontaneous hemorrhage, and inadequate blood supply (1). With its concomitant cardiovascular and cerebrovascular diseases, patients of stroke typically have poor prognosis and outcomes (2, 3). Stroke has been recognized as the second most deadly threat and the second leading contributors to severe disability worldwide (4, 5). The global epidemiology of stroke has also not been optimistic over the past few decades. The incident cases of stroke were 12.2 million in 2019, among which, 62.4% were of ischemic stroke, while 27.9% were of intracerebral hemorrhage, and 9.7% were subarachnoid hemorrhage cases (6). Meanwhile, the lifetime risk of stroke is approximately 25% from the age of 25 among both men and women (6). In 2019, the deaths caused by stroke amounted to 6.55 million and the disability-adjusted life years (DALYs) of stroke patients also reached 143 million (6).

Clinically, there are many factors that can affect the prognosis of stroke, which in general can be mainly divided into basic information for patients, complications, subtypes of stroke, and the treatments (7–9). Many complications can have an impact on the prognosis of stroke, including atherosclerosis (10), diabetes mellitus (9, 11), atrial fibrillation (12), cerebral palsy (13), and some cancers (14). Some stroke subtypes interact with specific complications and lead to a deterioration in prognosis (15–17). For instance, certain coagulation defects can cause abnormal traumatic injuries with blood-brain barrier disruption and exacerbate risk of hemorrhagic stroke (18, 19). Stroke patients may also face difficult treatment choices, such as the controversy over the use of anticoagulants like warfarin in cases of ischemic stroke complicated by gastrointestinal bleeding (20–22). The influence of a multitude of factors leads to increased complexity in the identification and therapeutic management of stroke patient prognosis.

Several previous studies used machine learning to solve problems related to stroke and other diseases. Cheon et al. used a fully connected neural network (FCNN) to identify factors affecting stroke mortality and had an AUC of 0.8. However, their principal component analysis (PCA) was not clinically interpretable (23). Heo et al. built a model using machine learning to predict long-term outcomes in acute stroke, but they only used six variables from the Analysis of Lausanne (ASTRAL) scores, which did not include comprehensive comorbidities and demographic factors (24). Ambale-Venkatesh et al. combined machine learning with deep phenotyping to improve the accuracy of cardiovascular event predictions (25). Some existing research generally lacks overall consideration of all comorbidities together and sometimes the model is not optimal.

Given the particular complexity and variety of contributing factors to stroke outcomes, which are difficult to predict globally using traditional research methods, such study limitations reduced predictive accuracy and limited to a comprehensive consideration of multiple factors. The aim of our study is to supplement and exploit multiple comorbidities, laboratory tests and demographic factors to more accurately predict death related to stroke, and furthermore, to make inferences about the heterogeneity of treatment in stroke patients to guide better treatment planning.

## 2. Materials and methods

### 2.1. Study design

We conducted a retrospective study of the risk factors for death in stroke patients and trained several machine learning (ML) models to predict their mortality during hospitalization and within 30 days after discharge. Patients with stroke were enrolled from the Medical Information Mart from Intensive Care (MIMIC)-IV version 2.0 (26), based on the International Classification of Diseases version 10 (ICD-10), a public dataset maintained by the Beth Israel Deaconess Medical Center. The period for study enrolment was from 2008 to 2019. The study was approved by online certification.

**Figure 1:**
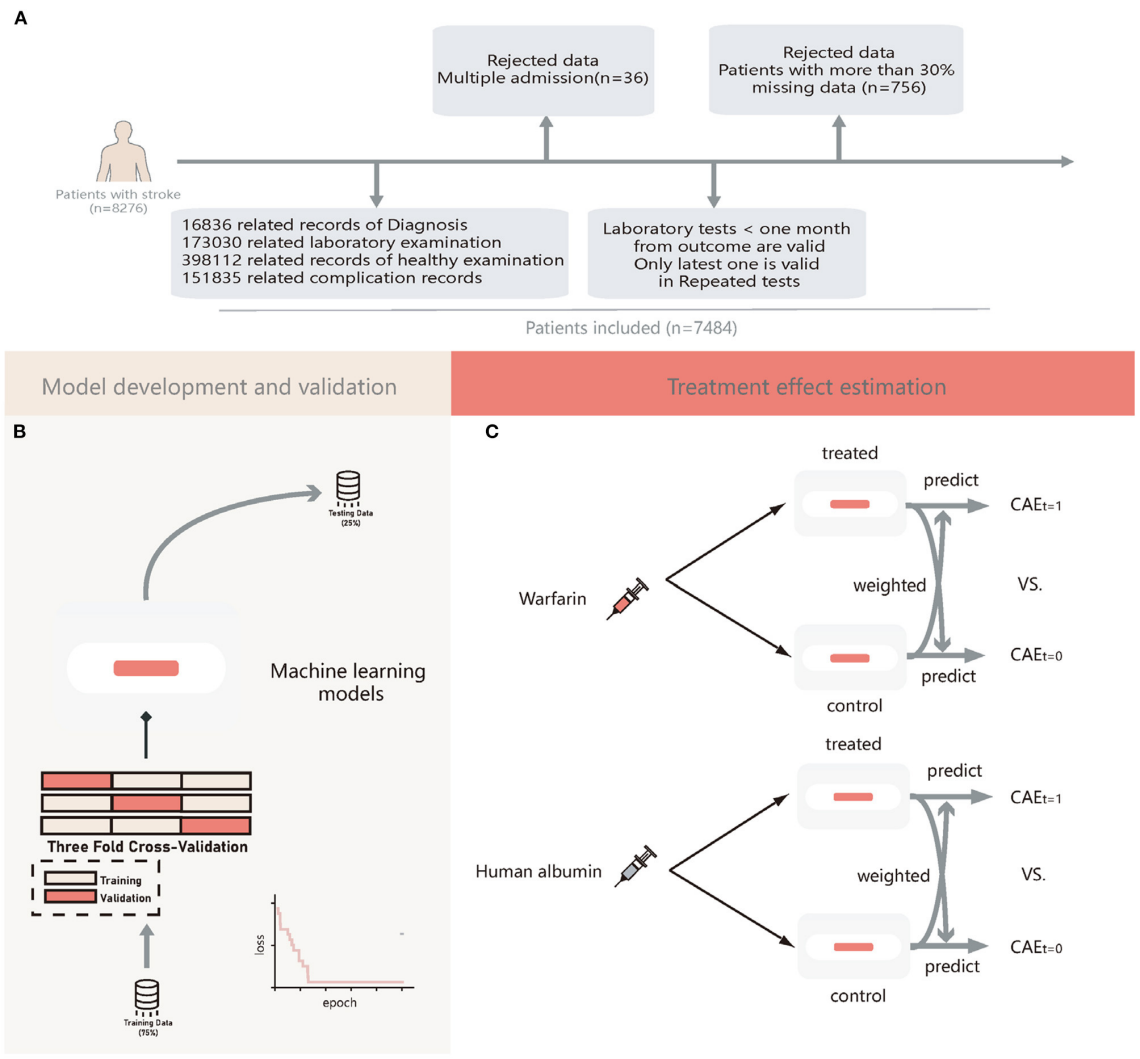
The flow chat of patients' enrollment, model development and validation, and treatment effect estimation. **(A)** Flow chart of patients' enrollment; **(B)** Flow chart machine learning models development and validation; **(C)** Flow chart of estimation of treatment heterogeneity effects. CATE, conditional average treatment effect; t = 1, patients treated with warfarin or human albumin; t = 0, patients not treated with warfarin or human albumin.

Patients diagnosed with stroke were included according to the ICD-10. We extracted a total of 8,276 patients with stroke, excluding 36 patients admitted to the hospital many times and 757 patients whose records did not contain adequate and relevant information within one month from the outcome (more than 30%). Finally, we extracted and sorted all the information of the remaining 7,483 patients, and a flowchart of patient selection and data collection is shown in Figure 1A.

### 2.2. Principle variables

The dependent variable was the mortality rate of stroke patients. Participants who died during hospitalization and within 30 days after discharge were considered deaths. The independent variables reflecting sociodemographic status included age, gender, marital status, race, insurance type, height, weight, and body weight index (BMI). Medical variables included the course of the disease, laboratory tests, and complications. Drug-related information included the usage of warfarin and human albumin, which was the most frequently used medicine for stroke patients in this database. We also included the detailed subtypes of stroke and whether the stroke was recurrent as independent variables. We excluded complications without a clear diagnosis to make our study reproducible and explainable. We obtained 145 variables for the ML model.

### 2.3. Machine learning algorithms and training strategy

We used six ML algorithms, which were Neural Oblivious Decision Ensemble (NODE) (27); CatBoost (28); XGBoost (29); LightGBM (30); fully connected neural network (FCNN); and logistic regression (LR).

NODE, a state-of-the-art Deep Learning (DL) model specialized for tabular data, uses oblivious decision trees (ODTs) (31) as weaker learners and inherits hierarchical representation and attention mechanism from neural networks. Every layer of NODE is densely connected to the original inputs and is trained end-to-end *via* backpropagation. The final prediction of NODE was obtained by averaging the outputs of all ODTs from all layers. We used Quasi-Hyperbolic Adam as an optimization strategy, which was recommended in the original paper (32). An FCNN is a common DL structure that contains several fully connected layers and uses ReLU as a nonlinear activation function. CatBoost is a gradient-boosted decision tree (GBDTs) model released in 2018 that also uses ODTs as weaker learners. The other two GBDTs models are XGBoost and LightGBM. We also used LR for comparison.

All patients were randomly allocated to a testing set of 25% samples unseen in the model development and used to evaluate the final model performance. A training set of 75% of the samples was used for building the model. During the training period, we used 3-folds-cross-validation to tune the model hyperparameters; for each time, the model trained on two-thirds of the training set and validated on the remaining one-third of the training set. For DL models, the loss of each step was recorded. The training was terminated automatically if it did not decrease in 1,000 iterations. For GBDTs, we used a random search algorithm to obtain the best models. A flowchart of the model development is shown in Figure 1B. We used the median to fill in missing values.

### 2.4. Estimation of treatment heterogeneity effects

We further estimated the therapeutic effects of human albumin and warfarin in individual patients using Meta-learner (33). It was a three-stage estimation, in which two GBDTs were used in the first stage to estimate the conditional average treatment effects (CATE) for the treatment and control groups separately, followed by estimation of the control group outcome using a GBDT built on the treatment group and estimation of the treatment group outcome using another one built on the control group, and finally estimation of the final CATE was weighted by the estimates obtained in the second stage. This leads to a more causal inference for individual treatment effects (ITE).

ITE was defined as the outcome estimation of a patient receiving human albumin or warfarin minus the outcome estimation for the same patient not receiving human albumin or warfarin. We then divided patients into consistent (Consis.) and inconsistent (In-consis.) groups in the testing set based on the actual treatment they received and the ITE values. This process was illustrated in Figure 1C.

### 2.5. Statistical analysis

PostgreSQL was used to extract and store the data from MIMIC-IV. All statistical analyses were performed using R, continuous variables were reported as the median and interquartile range (IQR), and categorical variables were presented as numbers and percentages (%). To compare continuous variables between the two groups, we used the Welch *t*-test and Mann–Whitney *U*-test, as appropriate. The chi-square test and Fisher's exact test were used to compare categorical variables, as appropriate.

## 3. Results

### 3.1. Demographic results

A total of 7,483 participants were included in this study, with 6,069 and 1,414 participants in the control and death groups, respectively. The median age was 69.0 years (59.0–79.0 years), and 50.3% of the patients were male. The total mortality rate was 18.9% (95% CI 18.0–19.8%).

A comparison of demographic status is shown in Table 1. The death group was older than the control group (74.0 vs. 68.0, *p* < 0.001). Most of the participants in the control group were married (45.6%, 95% CI 44.3–46.9%), whose proportion was higher than that of the death group (37.6%, 95% CI 35.1–40.2%). The proportion of single and divorced showed the same traction. However, the proportion of widowed individuals showed an opposite trend. The proportion of widowed individuals was 20.9% (95% CI 18.8–23.1%) in the death group and 14.5% (95% CI 13.6–15.4%) in the control group. The body mass index (BMI) of the death group was lower than that of the control group (26.3 vs. 27.3, *p* < 0.001). No statistically significant differences were found in sex or race.

**Table 1:** Comparison of demographic status.

	**Control**	**Dead**	***p-*value**
Age, median (IQR), y	68.0 (57.0–77.0)	74.0 (64.0–83.0)	<0.001^**^
BMI, median (IQR)	27.3 (23.8–31.4)	26.3 (22.9–30.3)	<0.001^**^
Sex			0.5136
Male	3042 (50.1%)	723 (51.1%)	
Female	3027 (49.9%)	691 (48.9%)	
Race			0.505
Asian	212 (3.5%)	60 (4.3%)	
Black	801 (13.2%)	194 (13.7%)	
White	3975 (65.6%)	926 (65.6%)	
Latin	227 (3.7%)	46 (3.3%)	
Other race	844 (13.9%)	185 (13.1%)	
Insurance type			<0.001^**^
Medicare	2896 (47.7%)	796 (56.3%)	
Other insurance	2906 (47.9%)	563 (39.8%)	
Medicaid	267 (4.4%)	55 (3.9%)	
Marital status			<0.001^**^
Married	2767 (45.6%)	532 (37.6%)	
Widowed	878 (14.5%)	295 (20.9%)	
Single	1492 (24.6%)	285 (20.2%)	
Divorced	537 (8.8%)	92 (6.5%)	

Other races, including unknown, unable to obtain, and multiple races; other insurance, including no insurance, employer-based insurance plan, and individual health insurance. Statistical analysis: ^**^p < 0.01.

### 3.2. Model predictive performance

**Figure 2:**
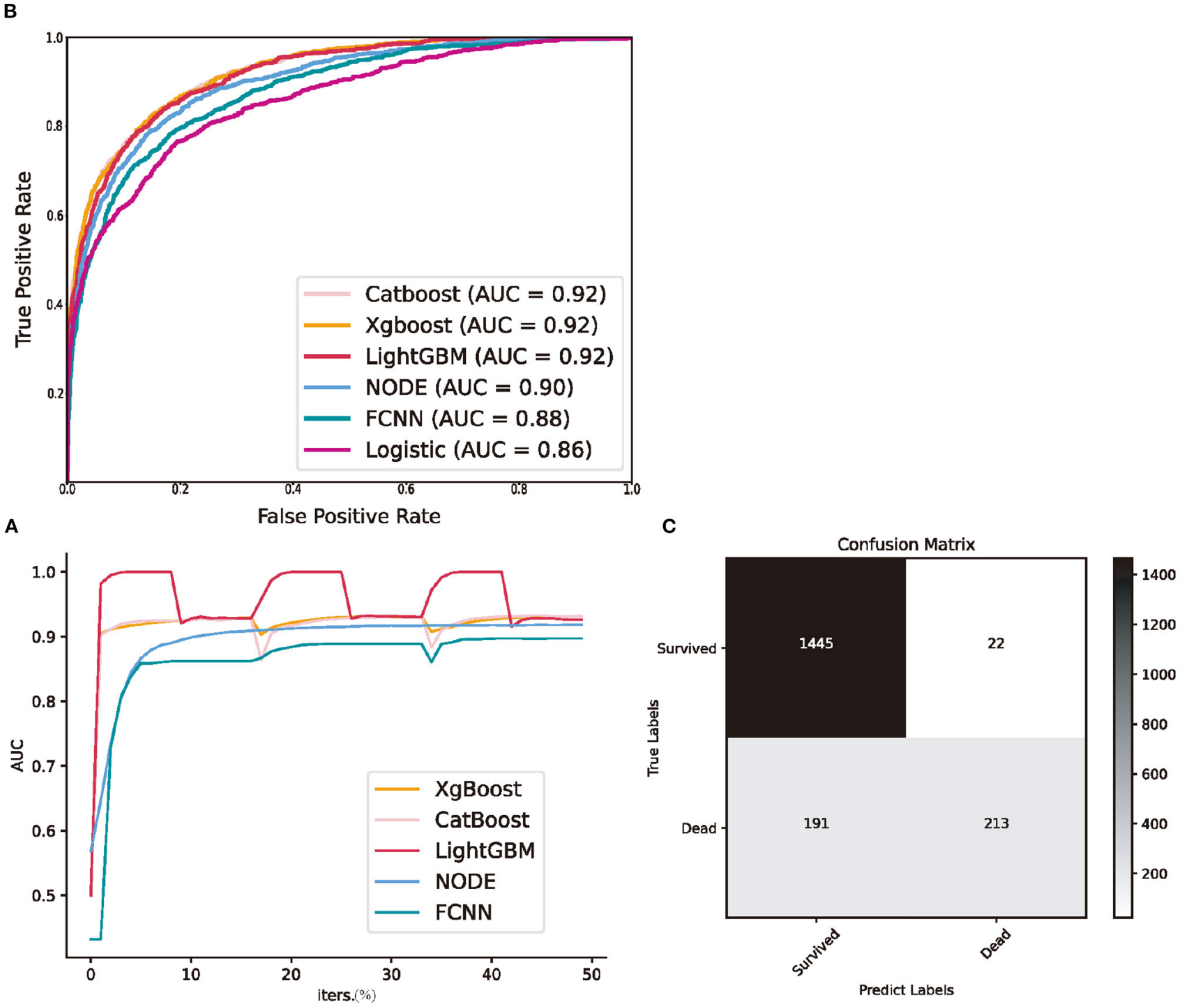
Predictive performance of machine learning models in the testing set. **(A)** the validation area under receiver operating characteristic curve during three folds cross validation; **(B)** receiver operating characteristic curve of all machine learning models; **(C)** the confusion matrix of CatBoost. NODE, Neural Oblivious Decision Ensembles; FCNN, fully connected neural network; Logistic, logistic regression; AUC, area under receiver operating characteristic curve; iters., iterations.

We calculated accuracy (ACC.), the area under the receiver operating characteristic curve (AUC), which is the ability to weigh true positives and false positives, precision score (Prec.), and F-measure (F1), which is a comprehensive indicator reflecting the true positive rate and sensitivity rate. The validation AUC curve during the training period is shown in Figure 2A, which exhibits oscillation owing to 3-fold cross-validation. The predictive performance of each model is presented in Table 2. The performances of GBDTs and NODE were close to acceptable levels. CatBoost has the highest ACC., Prec., and F1 (ACC: 0.8993 [0.8972–0.9014]; Prec.: 0.8155 [0.8072–0.8214]; AUC: 0.9217 [0.9188–0.9238]; F1: 0.6805 [0.6735–0.6855]), XGBoost has the highest F1 (ACC.: 0.8969 [0.8955–0.8987]; Prec.: 0.7783 [0.7689–0.7841]; AUC: 0.9175 [0.9153–0.9194]; F1: 0.6890 [0.6824–0.6939]). However, FCNN performed worse than GBDTs in ACC., AUC, and F1 (ACC.: 0.8726 [0.8699–0.8744]; Prec.: 0.8129 [0.8021–0.8227]; AUC: 0.8796 [0.8763–0.8832]; F1: 0.5328 [0.5231–0.5391]). LR has the lowest Prec. and AUC (ACC.: 0.8753 [0.8729–0.8773]; Prec.: 0.7298 [0.7206–0.7372]; AUC: 0.8591 [0.8555–0.8622]; F1: 0.6003 [0.5923–0.6069]). Additionally, we demonstrated the receiver operating characteristic (ROC) curve in Figure 2B.

**Table 2:** Predictive performance of each model.

**Model**	**ACC. [95% CI]**	**Prec. [95% CI]**	**AUC [95% CI]**	**F1 [95% CI]**
NODE	0.8857 [0.8833–0.8874]	0.7368 [0.7274–0.7432]	0.9008 [0.8975–0.9029]	0.6528 [0.6455–0.6580]
FCNN	0.8726 [0.8699–0.8744]	0.8129 [0.8021–0.8227]	0.8796 [0.8763–0.8832]	0.5328 [0.5231–0.5391]
XGBoost	0.8969 [0.8955–0.8987]	0.7783 [0.7689–0.7841]	0.9175 [0.9153–0.9194]	**0.6890 [0.6824–0.6939]**
CatBoost	**0.8993 [0.8972–0.9014]**	**0.8155 [0.8072–0.8214]**	**0.9217 [0.9188–0.9238]**	0.6805 [0.6735–0.6855]
LightGBM	0.8955 [0.8931–0.8972]	0.7753 [0.7657–0.7827]	0.9086 [0.9059–0.9106]	0.6804 [0.6730–0.6854]
LR	0.8753 [0.8729–0.8773]	0.7298 [0.7206–0.7372]	0.8591 [0.8555–0.8622]	0.6003 [0.5923–0.6069]

ACC., accuracy; AUC, the area under the receiver operating characteristic curve; Prec., precision score; F1, F-measure. NODE, Neural Oblivious Decision Ensemble; FCNN, fully connected neural network; LR, Logistic Regression Model. Bolded font indicates the best indicator among all models.

### 3.3. Recognition of the heterogenic treatment effects

**Figure 3:**
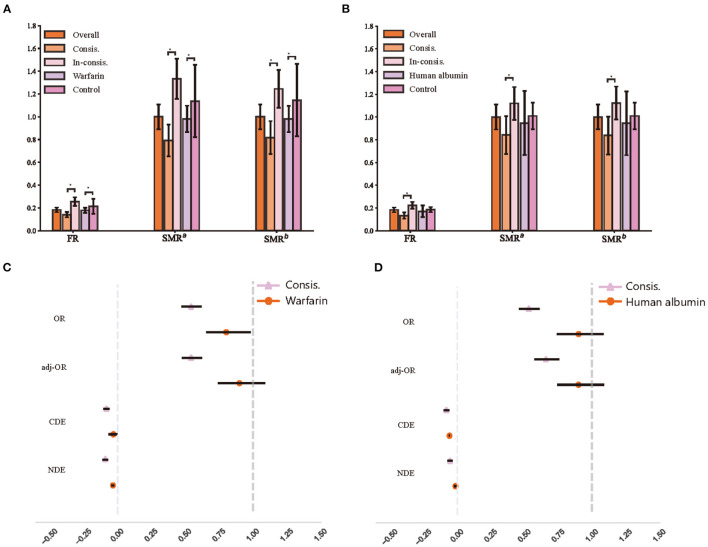
The recognition of treatment heterogeneity effects. **(A)** Fatality of different warfarin treatment groups; **(B)** Fatality of different human albumin treatment groups; **(C)** The average treatment effects of warfarin treatment groups; **(D)** The average treatment effects of human albumin treatment groups. FR, fatality rate; SMR, standardized mortality rate; SMR^a^, standardized mortality rate that controls age and sex; SMR^b^, standardized mortality rate that controls age, sex, and all subtypes of stroke; OR, odds ratio; adj-OR, odds ratio that adjusted with augmented inverse probability weighting; CDE, controlled direct effect; NDE, natural direct effect.

We presented the fatality rates (FR) of the treatment group, control group, Consis. group and In-consis. group of warfarin and human albumin in Figures 3A, C, respectively. We also demonstrated their average treatment effects (ATE), which was the Figures 3B, D. In the calculation of the ATE of the factor that whether the patient's actual treatment is in line with ITE (Consis.), treatment was considered as a mediator and was controlled. Meanwhile, subtypes of stroke, recurrent stroke, age, and sex were considered as confounders in the calculation of standardized mortality rate (SMR) and ATE for both Consis. and treatment. In the estimation of ATE, we used augmented inverse probability weighting (AIPW) (34) to correct the OR values. Additionally, we calculated controlled direct effects (CDE) and natural direct effects (NDE) (35), in which, CDE measures whether a specific patient's outcome would have improved if they had been treated (or be in Consis.) when the confounders hold at a predetermined level, while NDE holds confounders fixed in the same level under untreated condition. The CDE and NDE were presented as the slope of a linear regression.

In the testing set (1,871 of patients), 1,706 (91.2%) patients have been taken warfarin while the treatment was deemed appropriate for 987 (57.3% of treated with warfarin) patients, and 96 (58.2% of non-treated with warfarin) patients were considered should be on warfarin. 294 (15.7%) patients were in human albumin treatment group; 821 (52.1% of non-treated with human albumin) patients were considered by the model that should be treated.

We observed a lower fatality rate (FR) in the Consis. than in the In-consis. (Consis vs. In-consis: in warfarin, 14.1% vs. 25.6%, *p* < 0.0001; in human albumin, 13.3% vs. 22.4%, *p* < 0.0001). Significant differences remained after correction for confounders (SMR^b^ of Consis: in warfarin, 0.82, 95% CI: 0.75–0.89; in human albumin, 0.84, 95% CI: 0.76–0.93). The odds ratio (OR), AIPW adjusted OR (adj-OR), CDE, and NDE of Consis. remained significant and lower than treatment, except for CDE of human albumin (Consis.: −0.08, 95% CI: −0.10 to −0.06); human albumin: −0.06). The adj-OR of Consis. in warfarin was 0.54 (0.47–0.62) and that of human albumin was 0.66 (0.57–0.76), indicating a strong protective factor. The CDE and NDE also showed that Consis. had a direct effect on outcome (unaffected by treatment ratio and other confounders).

### 3.4. Model interpretation

**Figure 4:**
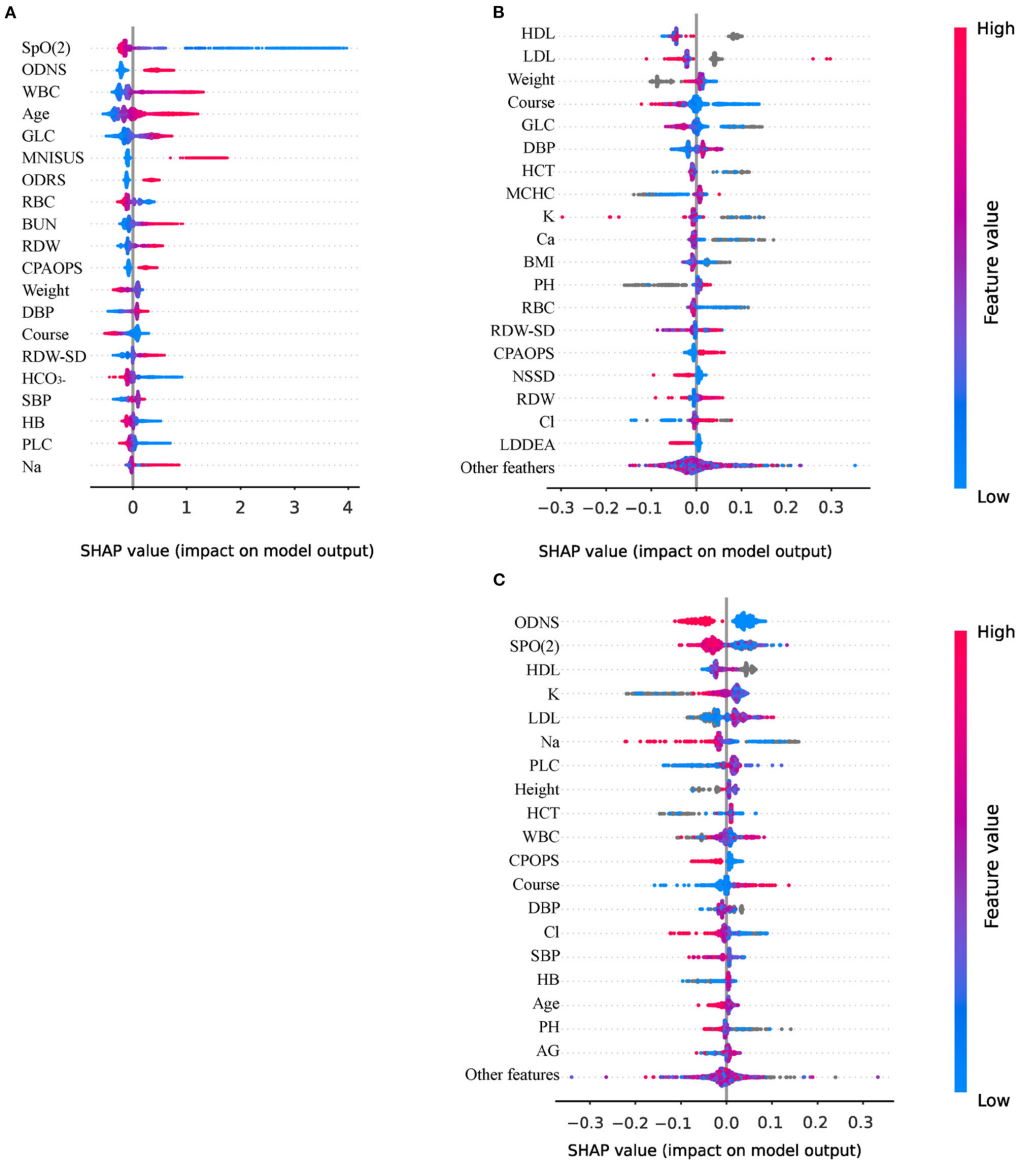
Interpretation of models using Shapley Additive Explanations (SHAP) analysis. **(A)** The variables importance of CatBoost; **(B)** The importance of variables to explain warfarin treatment heterogeneity; **(C)** The importance of variables to explain human albumin treatment heterogeneity. ODNS, other disorders of the nervous system; WBC, white blood cell count; GLC, blood glucose; MNISUS, malignant neoplasms of ill-defined, secondary and unspecified sites; ODRS, other diseases of the respiratory system; RBC, red blood cell count; BUN, urea nitrogen; RDW, red blood cell distribution width; CPAOPS, cerebral palsy and other paralytic syndromes; DBP, diastolic blood pressure; RDW-SD, standard deviation of red blood cell distribution width; HCO_3−_, blood bicarbonate; SBP, Systolic pressure; HB, hemoglobin; PLC, platelet count; Na, blood natrium; HCT, hematocrit; MCHC, mean red blood cell hemoglobin concentration; Ca, serum calcium; K, blood potassium; NSSD, neurotic, stress-related and somatoform disorders; LDDEA, lung diseases due to external agents; Cl, blood chloride; HDL, high-density lipoprotein; LDL, low-density lipoprotein; AG, anion gap, PH; phosphoric acid.

CatBoost exhibits the highest ACC., Prec., and F1. Thus, we conducted a Shapley Additive Explanations (SHAP) analysis to reveal the distribution of the effect of each input acting on CatBoost. Figure 4A shows a SHAP summary plot sorted by the feature importance of the top 20 important features, wherein every point represents a sample, and the horizontal coordinate is the SHAP value of each feature. A higher intensity of red indicates a higher feature value, while a higher intensity of blue indicates a lower feature value.

First, the lower the oxygen saturation (SpO_2_), the higher the SHAP value, which means that the patient is more likely to die. The protective factors were red blood cell count (RBC), followed by weight, course of disease, and blood bicarbonate (HCO_3−_). The most important risk factor was other disorders of the nervous system (ODNS), followed by white blood cell count (WBC), age, glucose (GLC), and malignant neoplasms of ill-defined, secondary and unspecified sites (MNISUS). We also presented the SHAP plot of Meta-learner of warfarin (Figure 4B) and human albumin (Figure 4C) that indicated which variables more significantly affect the inference of heterogeneity of treatment.

## 4. Discussion

Stroke remains one of the most destructive and prevalent nervous system diseases worldwide, responsible for disability or death in many individuals every year and a significant increase in DALY (36, 37). The importance of our study in the context of stroke mortality is that the features recognized in this study can identify the factors significantly related to mortality and treatment effects using statistical methods and ML models.

We analyzed the distribution of the demographic factors included in the study. Elderly individuals are more likely to have a stroke and a serious clinical outcome (38), which is consistent with our study. We also found that patients with higher BMI were more likely to survive after stroke (control vs. death, 26.3 vs. 27.3, *p* < 0.001), however, this association may depend on age (39). Additionally, we found more patients with other insurance types in the control group than in the death group [control vs. death, 47.9 vs. 39.8%, 95% CI (control), 46.6–49.1%]. Other insurance statuses include no insurance, employer-based insurance plans, and individual health insurance, most of which are commercial insurance, which may be related to a better economic level.

This study showed that the use of the ML method helps predict death after a stroke. To the best of our knowledge, this study achieved the highest AUC (AUC: 0.9217 [0.9188–0.9238]). Previous studies have suggested that FCNN or Deep Neural Network (DNN) is the best model for predicting post-stroke mortality and outperforms other traditional ML models (23, 24). However, in our study, the FCNN performed the worse than GBDTs and NODE (ACC.: 0.8857 [0.8833–0.8874]; AUC: 0.9008 [0.8975–0.9029]). The ML model with the highest performance was CatBoost and the rest of the GBDTs achieved high performance. Tree-based models appear more suitable for structured medical data, regardless of whether the model is implemented with ensemble methods, such as GBDTs, or layer-wise structures, such as NODE. However, GBDTs and DL models have their own advantages. GBDTs can help achieve relatively high accuracy in a very short period. DL models are fully differentiable and scalable, which means that researchers can arbitrarily change their model structure to fit the data better.

We further used meta-learner to identify heterogeneous treatment effects in the stroke population. We observed that 55.6% of the warfarin current use (or not use) and 52.1% of the status quo human albumin treatment (or control) in the testing set were considered inappropriate by model. Even after controlling for treatment factors, demographic factors and subtypes, survival outcomes were still significantly better in those who were consistent with the model judgments than in those who were inconsistent. Extrapolation of treatment effects for the population level does not necessarily hold in individual patients (34) and treatment heterogeneity has been reported to exist in stroke patients (40). However, studies of ITE in stroke patients are scarce (41) and, to our best knowledge, there is no such discussion of warfarin and human albumin, which are common drugs (42–45). Our study shows that ML can be used to help identify individuals with heterogeneous responses to treatment in stroke patients and thus make better treatment plans.

ML is a good predictive tool and usually has high accuracy. However, it has always been regarded as a “black box,” indicating poor interpretability. In our study, we conducted SHAP analysis to interpret one of our best models and obtain several risk and protective factors to help better understand the role of various factors in post-stroke mortality. Most of the results were consistent with clinical knowledge (46–49). In addition, we showed the 20 variables considered the most important by the model for the estimation of treatment heterogeneity, mostly laboratory indicators, which are worthy of further investigation. These results can be referenced in subsequent studies as a screening of important variables to narrow the scope.

In summary, we used several highly interpretive machine learning models to predict stroke prognosis with the highest accuracy to date and to identify heterogeneous treatment effects of warfarin and human albumin in stroke patients. Our interpretation of the model yielded a number of findings that are consistent with clinical knowledge and warrant further study and verification.

Our study has some limitations. The data we used included only inpatients from one hospital. These inpatients are already affected by stroke and usually have more serious conditions than the average population of stroke patients (50, 51). This narrow scope may limit the general applicability of our results. Since this is the first study to use machine learning to analyze such a wide range of variables in a population that has complex comorbidity factors, such as simultaneous hemorrhagic stroke and ischemic stroke [839 (11.2%)], we did not perform further analysis and inferences on all the conclusions obtained. In further studies, we will explore in depth the factors affecting survival or treatment effects and group subtypes of stroke to draw even further conclusions.

## Data availability statement

The original contributions presented in the study are included in the article/supplementary material, further inquiries can be directed to the corresponding author.

## Ethics statement

The studies involving human participants were approved by the Massachusetts Institute of Technology (Cambridge, MA) and Beth Israel Deaconess Medical Center (Boston, MA). Written informed consent for participation was not required for this study in accordance with the national legislation and the institutional requirements.

## Author contributions

EZ: experimental design, data analysis, model development, and manuscript writing. ZC: data acquisition, data analysis, and manuscript writing. PA: experimental design, data analysis, and manuscript writing. JW: experimental design and manuscript writing. MZ and ZX: data analysis and model development. JL: data analysis and manuscript revise. ZA: experimental design and manuscript revise. All authors contributed to the article and approved the submitted version.

## References

[1] Hankey GJ Blacker DJ. Is it a stroke? BMJ. (2015) 350:h56. 10.1136/bmj.h56 25591946

[2] Benjamin EJ Virani SS Callaway CW Chamberlain AM Chang AR Cheng S. Heart disease and stroke statistics-2018 update: a report from the american heart association. Circulation. (2018) 137:e67–e492. 10.1161/CIR.0000000000000573 29386200

[3] Stinear CM Lang CE Zeiler S Byblow WD. Advances and challenges in stroke rehabilitation. Lancet Neurol. (2020) 19:348–60. 10.1016/S1474-4422(19)30415-6 32004440

[4] Timmis A Vardas P Townsend N Torbica A Katus H De Smedt D. European society of cardiology: cardiovascular disease statistics 2021. Eur Heart J. (2022) 43:716–99. 10.1093/eurheartj/ehab892 35016208

[5] Organization WH. World health statistics 2021. Geneva: World Health Organization (2021).

[6] Global regional and and national burden of stroke and its risk factors 1990-2019: 1990-2019: a systematic analysis for the global burden of disease study 2019. Lancet Neurol. (2021) 20:795–820. 10.1016/S1474-4422(21)00252-0 34487721 PMC8443449

[7] Campbell BCV Khatri P. Stroke. Lancet. (2020) 396:129–42. 10.1016/S0140-6736(20)31179-X 32653056

[8] Cipolla MJ Liebeskind DS Chan SL. The importance of comorbidities in ischemic stroke: impact of hypertension on the cerebral circulation. J Cereb Blood Flow Metab. (2018) 38:2129–49. 10.1177/0271678X18800589 30198826 PMC6282213

[9] Alloubani A Saleh A Abdelhafiz I. Hypertension and diabetes mellitus as a predictive risk factors for stroke. Diabetes Metab Syndr. (2018) 12:577–84. 10.1016/j.dsx.2018.03.009 29571978

[10] Virani SS Alonso A Aparicio HJ Benjamin EJ Bittencourt MS Callaway CW. Heart disease and stroke statistics-2021 update: a report from the american heart association. Circulation. (2021) 143:e254–743. 10.1161/CIR.0000000000000950 33501848 PMC13036842

[11] Dardiotis E Aloizou AM Markoula S Siokas V Tsarouhas K Tzanakakis G. Cancer-associated stroke: Pathophysiology, detection and management (Review). Int J Oncol. (2019) 54:779–96. 10.3892/ijo.2019.4669 30628661 PMC6365034

[12] Seiffge DJ Werring DJ Paciaroni M Dawson J Warach S Milling TJ. Timing of anticoagulation after recent ischaemic stroke in patients with atrial fibrillation. Lancet Neurol. (2019) 18:117–26. 10.1016/S1474-4422(18)30356-9 30415934 PMC6524642

[13] Dunbar M Kirton A. Perinatal stroke: mechanisms, management, and outcomes of early cerebrovascular brain injury. Lancet Child Adolesc Health. (2018) 2:666–76. 10.1016/S2352-4642(18)30173-1 30119760

[14] Navi BB Kasner SE Elkind MSV Cushman M Bang OY DeAngelis LM. Cancer and embolic stroke of undetermined source. Stroke. (2021) 52:1121–30. 10.1161/STROKEAHA.120.032002 33504187 PMC7902455

[15] Maida CD Norrito RL Daidone M Tuttolomondo A Pinto A. Neuroinflammatory mechanisms in ischemic stroke: focus on cardioembolic stroke, background, and therapeutic approaches. Int J Mol Sci. (2020) 21:454. 10.3390/ijms21186454 32899616 PMC7555650

[16] Petersen MA Ryu JK Akassoglou K. Fibrinogen in neurological diseases: mechanisms, imaging and therapeutics. Nat Rev Neurosci. (2018) 19:283–301. 10.1038/nrn.2018.13 29618808 PMC6743980

[17] Feske SK. Ischemic stroke. Am J Med. (2021) 134:1457–64. 10.1016/j.amjmed.2021.07.027 34454905

[18] Unnithan AKA Mehta P. Hemorrhagic Stroke. Treasure Island, FL: StatPearls Publishing (2011). 32644599

[19] Meyfroidt G Bouzat P Casaer MP Chesnut R Hamada SR Helbok R. Management of moderate to severe traumatic brain injury: an update for the intensivist. Intensive Care Med. (2022) 48:649–66. 10.1007/s00134-022-06702-4 35595999

[20] Abraham NS Barkun AN Sauer BG Douketis J Laine L Noseworthy PA. American college of gastroenterology-canadian association of gastroenterology clinical practice guideline: management of anticoagulants and antiplatelets during acute gastrointestinal bleeding and the periendoscopic period. J Can Assoc Gastroenterol. (2022) 5:100–1. 10.1093/jcag/gwac010 35368325 PMC8972207

[21] Abrignani MG Gatta L Gabrielli D Milazzo G De Francesco V De Luca L. Gastroprotection in patients on antiplatelet and/or anticoagulant therapy: a position paper of national association of hospital cardiologists (ANMCO) and the italian association of hospital gastroenterologists and endoscopists (AIGO). Eur J Intern Med. (2021) 85:1–13. 10.1016/j.ejim.2020.11.014 33279389

[22] Carnicelli AP Hong H Connolly SJ Eikelboom J Giugliano RP Morrow DA. Direct oral anticoagulants vs. warfarin in patients with atrial fibrillation: patient-level network meta-analyses of randomized clinical trials with interaction testing by age and sex. Circulation. (2022) 145:242–55. 10.1161/CIR.0000000000001058 34985309 PMC8800560

[23] Cheon S Kim J Lim J. The use of deep learning to predict stroke patient mortality. Int J Environ Res Public Health. (2019) 16:1876. 10.3390/ijerph16111876 31141892 PMC6603534

[24] Heo J Yoon JG Park H Kim YD Nam HS Heo JH. Machine learning-based model for prediction of outcomes in acute stroke. Stroke. (2019) 50:1263–5. 10.1161/STROKEAHA.118.024293 30890116

[25] Ambale-Venkatesh B Yang X Wu CO Liu K Hundley WG McClelland R. cardiovascular event prediction by machine learning: the multi-ethnic study of atherosclerosis. Circ Res. (2017) 121:1092–101. 10.1161/CIRCRESAHA.117.311312 28794054 PMC5640485

[26] Johnson A Bulgarelli L Pollard T Horng S Celi LA Mark R. MIMIC-IV (version 20). (2022).

[27] Popov S Morozov S Babenko A. Neural Oblivious Decision Ensembles for Deep Learning on Tabular Data (2020).

[28] Dorogush AV Gulin A Gusev G Kazeev N Ostroumova L Vorobev A. Fighting biases with dynamic boosting. (2017).

[29] Chen T Guestrin C. “XGBoost: a scalable tree boosting system,” in Proceedings of the 22nd ACM SIGKDD International Conference on Knowledge Discovery and Data Mining. (2016).32561836

[30] Guolin Ke QM Thomas F Taifeng W Wei C Weidong M Qiwei Y. LightGBM: A highly efficient gradient boosting decision tree. (2017).

[31] Kohavi R. Bottom-up induction of oblivious read-once decision graphs. In: National Conference on Artificial Intelligence. (1994). Available online at: https://www.xueshufan.com/publication/2114154044

[32] Ma J Yarats D. Quasi-hyperbolic momentum and Adam for deep learning. arXiv [Preprint]. (2018). arXiv: 1810.06801. Available online at: https://www.researchgate.net/publication/328332350_Quasi-hyperbolic_momentum_and_Adam_for_deep_learning

[33] Künzel SR Sekhon JS Bickel PJ Yu B. Metalearners for estimating heterogeneous treatment effects using machine learning. Proc Nat Acad Sci. (2017) 116:4156–65. 10.1073/pnas.1804597116 30770453 PMC6410831

[34] Yao L Chu Z Li S Li Y Gao J Zhang A. A survey on causal inference. ACM arXiv [Preprint]. (2020) arXiv: 2002.02770. 15:1–46. 10.48550/arXiv.2002.02770

[35] Hu Y Li S Wager S. Average direct and indirect causal effects under interference. arXiv [Preprint]. (2021). arXiv: 2104.03802v4. Available online at: https://arxiv.org/abs/2104.03802v4

[36] Zhou M Wang H Zeng X Yin P Zhu J Chen W. Mortality, morbidity, and risk factors in China and its provinces, 1990-2017: a systematic analysis for the global burden of disease study 2017. Lancet. (2019) 394:1145–58. 10.1016/S0140-6736(19)30427-1 31248666 PMC6891889

[37] Feigin VL Forouzanfar MH Krishnamurthi R Mensah GA Connor M Bennett DA. Global and regional burden of stroke during 1990-2010: findings from the Global Burden of Disease Study 2010. Lancet. (2014) 383:245–54. 10.1016/S0140-6736(13)61953-4 24449944 PMC4181600

[38] Liu Q Wang X Wang Y Wang C Zhao X Liu L. Association between marriage and outcomes in patients with acute ischemic stroke. J Neurol. (2018) 265:942–8. 10.1007/s00415-018-8793-z 29464375 PMC5878185

[39] Dehlendorff C Andersen KK Olsen TS. Body mass index and death by stroke: no obesity paradox. JAMA Neurol. (2014) 71:978–84. 10.1001/jamaneurol.2014.1017 24886975

[40] Kent DM Saver JL Kasner SE Nelson J Carroll JD Chatellier G. Heterogeneity of treatment effects in an analysis of pooled individual patient data from randomized trials of device closure of patent foramen ovale after stroke. JAMA. (2021) 326:2277–86. 10.1001/jama.2021.20956 34905030 PMC8672231

[41] Vliet Pv Carey L Nilsson M. Targeting stroke treatment to the individual. Int J Stroke. (2012) 7:480–1. 10.1111/j.1747-4949.2012.00867.x 22805575

[42] Hankey GJ. Stroke. Lancet. (2017) 389:641–54. 10.1016/S0140-6736(16)30962-X 27637676

[43] Ruff CT Giugliano RP Braunwald E Hoffman EB Deenadayalu N Ezekowitz MD. Comparison of the efficacy and safety of new oral anticoagulants with warfarin in patients with atrial fibrillation: a meta-analysis of randomised trials. Lancet. (2014) 383:955–62. 10.1016/S0140-6736(13)62343-0 24315724

[44] Belayev L Liu Y Zhao W Busto R Ginsberg MD. Human albumin therapy of acute ischemic stroke: marked neuroprotective efficacy at moderate doses and with a broad therapeutic window. Stroke. (2001) 32:553–60. 10.1161/01.STR.32.2.553 11157196

[45] Lee SH Jang MU Kim Y Park SY Kim C Kim YJ. Effect of prestroke glycemic variability estimated glycated albumin on stroke severity and infarct volume in diabetic patients presenting with acute ischemic stroke. Front Endocrinol. (2020) 11:230. 10.3389/fendo.2020.00230 32373074 PMC7186307

[46] Feng GH Li HP Li QL Fu Y Huang RB. Red blood cell distribution width and ischaemic stroke. Stroke Vasc Neurol. (2017) 2:172–5. 10.1136/svn-2017-000071 28989807 PMC5628378

[47] Gu X Li Y Chen S Yang X Liu F Li Y. Association of lipids with ischemic and hemorrhagic stroke: a prospective cohort study among 267 500 chinese. Stroke. (2019) 50:3376–84. 10.1161/STROKEAHA.119.026402 31658904

[48] Potasso L Refardt J De Marchis GM Wiencierz A Wright PR Wagner B. Impact of sodium levels on functional outcomes in patients with stroke - a swiss stroke registry analysis. J Clin Endocrinol Metab. (2022) 107:e672–80. 10.1210/clinem/dgab650 34480576

[49] Appiah KO Minhas JS Robinson TG. Managing high blood pressure during acute ischemic stroke and intracerebral hemorrhage. Curr Opin Neurol. (2018) 31:8–13. 10.1097/WCO.0000000000000508 29076879

[50] Zahid S Ullah W Khan MZ Rai D Bandyopadhyay D Din MTU. Trends and outcomes of ischemic stroke after transcatheter aortic valve implantation, a US national propensity matched analysis. Curr Probl Cardiol. (2022) 47:100961. 10.1016/j.cpcardiol.2021.100961 34391762

[51] Shahjouei S Li J Koza E Abedi V Sadr AV Chen Q. Risk of subsequent stroke among patients receiving outpatient vs inpatient care for transient ischemic attack: a systematic review and meta-analysis. JAMA Netw Open. (2022) 5:e2136644. 10.1001/jamanetworkopen.2021.36644 34985520 PMC8733831

